# Apoptosis, Autophagy, and Pyroptosis: Immune Escape Strategies for Persistent Infection and Pathogenesis of Classical Swine Fever Virus

**DOI:** 10.3390/pathogens8040239

**Published:** 2019-11-16

**Authors:** Sheng-ming Ma, Qian Mao, Lin Yi, Ming-qiu Zhao, Jin-ding Chen

**Affiliations:** College of Veterinary Medicine; South China Agricultural University; Guangzhou 510642, China; mashengming@stu.scau.edu.cn (S.-m.M.); maoqian@stu.scau.edu.cn (Q.M.); yilin@scau.edu.cn (L.Y.); zmingqiu@scau.edu.cn (M.-q.Z.)

**Keywords:** classical swine fever virus, apoptosis, autophagy, pyroptosis, pathogenesis

## Abstract

Classical swine fever (CSF) is a severe acute infectious disease that results from classical swine fever virus (CSFV) infection, which leads to serious economic losses in the porcine industry worldwide. In recent years, numerous studies related to the immune escape mechanism of the persistent infection and pathogenesis of CSFV have been performed. Remarkably, several independent groups have reported that apoptosis, autophagy, and pyroptosis play a significant role in the occurrence and development of CSF, as well as in the immunological process. Apoptosis, autophagy, and pyroptosis are the fundamental biological processes that maintain normal homeostatic and metabolic function in eukaryotic organisms. In general, these three cellular biological processes are always understood as an immune defense response initiated by the organism after perceiving a pathogen infection. Nevertheless, several viruses, including CSFV and other common pathogens such as hepatitis C and influenza A, have evolved strategies for infection and replication using these three cellular biological process mechanisms. In this review, we summarize the known roles of apoptosis, autophagy, and pyroptosis in CSFV infection and how viruses manipulate these three cellular biological processes to evade the immune response.

## 1. Introduction

Classical swine fever (CSF) is a serious porcine disease driven by the CSF virus (CSFV) that results in fever, leukopenia, abortion, hemorrhage, and high mortality: it has brought substantial economic losses to the world pig industry and has been classified as a class A infectious disease by the World Organization for Animal Health (OIE) [1,2]. CSFV is a single-stranded RNA flavivirus in the *Pestivirus* genus with a 12.3-kb genome and has a tropism for vascular endothelial cells and immune system cells [3]. This virus encodes a single 3898 amino acid polyprotein in its open reading frame (ORF), and this protein in turn undergoes processing to yield four structural proteins (C, E^rns^, E1, and E2) and eight nonstructural proteins (N^pro^, P7, NS2, NS3, NS4A, NS4B, NS5A, and NS5B) [4]. Infection with highly virulent CSFV strains leads to the occurrence of typical CSF, with hemorrhagic syndrome and immunosuppression as the main features [5,6]. Currently, treatment options for CSF are still limited; instead, prevention with vaccines against CSFV is usually used [7,8]. However, under immune selection pressure, CSFV has evolved and developed mechanisms that escape the host immune response, resulting in an outbreak of CSF or establishing persistent infection in an immune flock [9,10,11]. Although many studies have investigated the interaction mechanism between CSFV and the host, the pathogenesis and immune escape mechanism of CSFV still remain unclear [12,13,14,15]. It is still necessary to explore the pathogenic mechanisms of CSFV in order to develop specific drugs and vaccines for effective CSF prevention, control, and eradication.

The occurrence, development, and outcome of infectious diseases are the result of interaction between pathogens and hosts. In the long-term struggle between host and virus, the host initiates different forms of cellular biological processes to restrict viral replication [16,17,18]. However, in order to achieve persistent infection, viruses have evolved a variety of mechanisms to regulate cellular biological processes, thereby affecting the host inflammatory response and even cell survival, thus avoiding the host antiviral immune response [19,20,21,22]. Importantly, apoptosis, autophagy, and pyroptosis are fundamental biological processes in both normal physiology and pathology [23,24,25,26]. Apoptosis, as the most thoroughly characterized form of programmed cell death, is a physiological cell death that occurs when multicellular organisms respond to endogenous or exogenous stimuli [23,27]. Autophagy is a cell survival mechanism that involves the degradation and recycling of cytoplasmic components, including long-lived proteins, protein aggregates, damaged cytoplasmic organelles, and intracellular pathogens [28]. Different from other forms of cell death in morphology and mechanics, pyroptosis is a proinflammatory form of cell death regulated by the inflammasome and caspase-1 activation [29]. All of these three cellular biological processes are an important part of the process of growth and development and tissue remodeling and immune regulation, and they play an important and complex role in the immune response to virus infection [23,24,25,26,27,28,29].

Like other members of the family of Flaviviridae viruses, CSFV is dependent on host cells for viral replication [30]. In the long-term struggle with CSFV, the host has evolved complex anti-infective mechanisms to protect itself from infection, such as apoptosis, autophagy, and pyroptosis [31,32,33,34]. At the same time, CSFV has also evolved to exploit these three cellular biological processes using various strategies as well as effective escape mechanisms [31,32,33,34]. In this review, we summarize the known molecular mechanisms through which CSFV induces apoptosis, autophagy, and pyroptosis and the association of these three cellular biological processes with the pathogenesis of CSFV.

## 2. Apoptosis in the Pathogenesis of CSFV

Apoptosis, also known as programmed cell death of type I, is a physiological cell death that occurs when multicellular organisms respond to endogenous or exogenous stimuli [23,27,35]. Abnormal cell apoptosis often leads to disease. The virus-induced apoptosis of host cells is one of the important mechanisms of viral pathogenesis [22]. Apoptosis is the first line of defense against viral infection in host cells [16]. The host cells quickly start the apoptotic process under the stimulation of viruses and restrict the replication and transmission of viruses by quickly clearing the infected cells [16,22,36]. During virus infection, apoptosis can be triggered by diverse cellular signals, including the death receptor-mediated extrinsic pathway, the intrinsic mitochondrial pathway, the granzyme B-mediated pathway, and the endoplasmic reticulum stress-mediated pathway. When cells induce apoptosis, they destroy the intracellular environment of virus replication and expose virus particles to the cellular immune environment. The exposed virus particles are quickly phagocytized by macrophages. Meanwhile, dendritic cells recognize virus-infected cells and function as antigens, cross-presenting to trigger antivirus immune responses. However, the apoptosis of immune cells is beneficial for the virus to escape the monitoring of the host immune system [37,38]. In addition, some viruses or viral components delay or inhibit cell apoptosis through some cellular regulatory mechanisms, thus achieving persistent infection and survival in host cells [39,40].

CSFV is a typical immunosuppressive disease that greatly harms the hematopoietic and immune systems [41]. As a single-stranded enveloped RNA virus, CSFV has a strong affinity for vascular endothelial cells and immune system cells [31]. Leukopenia, in particular lymphopenia, is a characteristic early event during CSFV. Leukopenia involves leukocyte subpopulations in a disparate manner, with B-lymphocytes, helper T-cells, and cytotoxic T-cells being the most affected [42]. High titers of CSFV have been detected in bone marrow at the early stage of virus infection, which led to necrosis and the apoptosis of bone marrow hematopoietic cells and the apoptosis of bone marrow lymphocyte [43]. These lines of evidence indicate that the decrease in T-lymphocytes in peripheral blood is closely related to the damage of bone marrow lymphocyte apoptosis caused by CSFV. During CSFV infection, both mature peripheral and bone marrow neutrophils are affected, whereas immature neutrophils increase absolutely in the periphery, as do (coincidentally) immature myeloid progenitors in the bone marrow [44]. Further research has found granulocytopenia and disrupted bone marrow function to be a result of the death of hematopoietic cells as a consequence of interactions between viral and host mechanisms. In one study that used Terminal-deoxynucleoitidyl Transferase Mediated Nick End Labeling (TUNEL) staining and immunohistochemical analyses to examine apoptosis, researchers observed that the number of apoptotic cells was greater than the number of CSFV-infected cells, with many apoptotic and nonapoptotic cells being positive for tumor necrosis factor alpha (TNF-α) staining. This thus suggested that CSFV can drive apoptosis directly and indirectly. Moreover, one or more factors expressed by CSFV-infected macrophages (e.g., TNF-α) may induce apoptosis in uninfected bystander cells [31]. CSFV envelope glycoprotein E^rns^ is important for the pathogenesis of CSFV. There is evidence that E^rns^ inhibits the concanavalin A-induced proliferation of porcine lymphocytes, and indeed, the apoptosis of lymphocytes has been detected after incubation with E^rns^ [45,46]. It is also believed that CSFV-infected cells secrete a large number of extracellular viral glycoprotein E^rns^, which induces apoptosis in adjacent noninfected CSFV cells. All these data suggest that the reduction of peripheral blood lymphocytes induced by CSFV infection may be the result of multiple mechanisms.

Interestingly, CSFV nonstructural protein N^pro^ and NS2 have also been shown to inhibit apoptosis. N^pro^ induces the proteasomal degradation of IRF3, thereby facilitating evasion of the interferon response, in addition to preventing the apoptotic death of cells in response to dsRNA, which may also be an important reason why CSFV infection in vitro cannot cause cytopathic effects [47]. Further investigations have showed that the interaction of N^pro^ and the antiapoptotic protein HAX-1 (HS-1-associated protein X-1) plays a prominent role in the regulation of apoptosis [48]. Moreover, CSFV NS2 activates the noncanonical nuclear factor-kappaB (NF-κB) transcription factor and induces endoplasmic reticulum stress in swine umbilical vein endothelial cells (SUVECs), thereby promoting the increased expression of interleukin (IL)-8 as well as of Bcl-2, which is an antiapoptotic protein [49,50]. SUVECs expressing green fluorescent protein (GFP)–NS2 were able to resist MG132-induced apoptosis [51]. This thus indicated that the mechanism for the CSFV NS2 protein inhibiting apoptosis may be an important process for the virus to achieve persistent infection.

In conclusion, the relationship between CSFV and apoptosis is complex. In the early stage of CSFV infection in some host cells, antiapoptotic effects are the main manifestation, which facilitate the replication of the virus in infected cells [47,48,49,50]. However, in the late stage of CSFV infection, apoptotic effects are predominant, which may be the defensive response of the body to viral infection or an important mechanism of severe damage to host tissue cells caused by viral infection [42,43,44,45]. 

## 3. Autophagy in the Pathogenesis of CSFV

Autophagy, also known as a cell survival mechanism, is a catabolic process that involves the degradation and recycling of cytoplasmic components, including long-lived proteins, protein aggregates, damaged cytoplasmic organelles, and intracellular pathogens [25,28]. During autophagy, cytoplasmic components are isolated by a membrane called the phagosome or isolation membrane, which expands to form a double-membrane vesicle called the autophagosome [51,52,53]. Autophagosomes can fuse with vesicles of the endocytic pathway to form amphisomes that eventually fuse with lysosomes where the sequestered material is degraded [25,52]. In eukaryotic cells, autophagy can be broadly classified as macroautophagy, microautophagy, and chaperone-mediated autophagy depending on the mechanism and molecular players involved in the targeting of a substrate to the lysosome [51]. Macroautophagy and microautophagy are relatively conservative in all eukaryotes, while chaperone-mediated autophagy generally occurs in higher eukaryotes [52]. In the presentation pathway, microautophagy and chaperone autophagy can directly present the target substance to lysosomes, while macroautophagy requires the formation of specific bilayer membrane complexes, which can be encapsulated and isolated for presentation [53,54]. Autophagy, as one of the innate immune mechanisms, can not only maintain cellular homeostasis, but also protect cells against the invasion of pathogenic microorganisms. Virus particles can be delivered to lysosomes for degradation [55]. In addition, autophagy can also activate cellular adaptive immunity by participating in antigen processing and presentation to major histocompatibility complex (MHC) class II molecules, thus affecting virus replication [56]. In *Sindbis virus* and *Tobacco mosaic virus* infections, autophagy successfully restricts intracellular pathogen replication and transmission [57,58]. Conversely, *human immunodeficiency virus type 1* and *herpes simplex virus type 1* can inhibit autophagy to facilitate replication [59,60]. Nevertheless, several viruses, such as *influenza A virus*, *dengue virus*, *hepatitis C virus*, and CSFV, have evolved strategies of replication using autophagic vesicles [32,61,62,63]. Autophagy is virus-specific; thus, understanding the interaction between autophagy and viral infection is essential to control disease transmission.

At present, several reports have shown that various Flaviviridae viruses, including the *Zika virus*, *dengue virus*, *West Nile virus*, *Japanese encephalitis virus*, and *hepatitis C virus*, activate and require some aspect of autophagy for robust viral replication [62,63,64,65,66]. CSFV, as an important member of the Flaviviridae family, has also been reported to manipulate autophagy during infection [32]. Pei et al. first performed the initial characterization of autophagy during CSFV infection in 2014 [32]. The authors showed that CSFV infection markedly elevated the number of double- and single-membrane vesicles in infected cells. These authors also showed that the virus infection not only induced both ATG12–ATG5 conjugation as well as the conversion of LC3-I to LC3-II, but also led to significant increases in ATG5 and BECN1 levels within cells infected with the CSFV virus. The authors also detected SQSTM1 degradation, indicating that CSFV infection enhanced autophagic flux and triggered a complete autophagic response. Moreover, CSFV resulted in increased CD63 and LC3 redistribution, with the colocalization of NS5A and E2 with LC3- and CD63-positive punctae. This thus suggested that these autophagosome-like structures may be necessary for the replication of CSFV. Conversely, no obvious changes in the expression level of autophagy marker proteins were present in mock- and UV-treated CSFV-infected cells. This thus suggested that UV irradiation disrupted the ability of CSFV to mediate autophagosome formation. They further showed that anti-E2 and anti-NS5A staining was primarily evident upon autophagosome-like vesicle membranes, suggesting that this is likely the site of CSFV replication. The authors also demonstrated that LC3 redistribution and the colocalization of LC3 and EGFP–NS5A occurred in pEGFP–NS5A-transfected cells. In addition, pEGFP–NS5A markedly increased the level of LC3-II and drove the degradation of SQSTM1. All of these findings not only enforced that CSFV replication is required for the induction of autophagy, but also suggested that NS5A is essential for the CSFV-mediated induction of autophagy. Importantly, these authors used autophagy regulators and shRNA to regulate the autophagic activities of CSFV-infected cells. The results explored that rapamycin treatment significantly upregulated viral E2 expression and resulted in increased CSFV yields, whereas 3-methyladenine (3-MA) treatment or shRNA-mediated knockdown of LC3 and BECN1 to inhibit autophagy reduced E2 protein levels and CSFV yields. The authors further found that modulating autophagy had a more significant impact on extracellular than intracellular virion yields. This thus indicated that autophagy is necessary both for viral replication and cytoplasmic virus relief.

Autophagy is divided into nonselective autophagy and selective autophagy according to the occurrence process. Under nutrient deficiency, cells maintain essential metabolic substances and energy by activating nonselective autophagy to degrade intracellular biomacromolecules and organelles [67]. On the contrary, selective autophagy usually occurs in the condition of adequate nutrition, which is a stress response of cells to remove damaged cells or overaccumulated proteins [68]. Mitophagy is a selective autophagy and an effective mitochondrial clearance mechanism in cells [69,70]. Many viral proteins can target mitochondria through their mitochondrial localization sequences during virus infection [71,72,73]. Damaged mitochondria caused by virus infection can be cleared up through mitophagy [69,70]. Previous studies have shown that CSFV infection induces the production of reactive oxygen species (ROS) in cultured host cells in vitro and leads to the disappearance of mitochondrial membrane potential in porcine peripheral blood lymphocytes, which is closely related to the reduction of mitochondrial numbers [74,75]. On this basis, Gou et al. performed a study to explore the mechanism of CSFV-induced mitophagy and the role of mitophagy in CSFV infection [34]. These authors showed that mitochondrial mass obviously decreased 36 h postinfection in PK-15 and 3D4/2 cells. Further, they treated cells with 3-MA, inhibiting phagophore formation and Bafilomycin A1 (BafA1), inhibiting the activity of vacuolar-type H+-ATPase before and during CSFV infection. These authors found that both autophagy inhibitors could invert the decline of mitochondrial mass induced by CSFV infection. Moreover, these authors observed that mitochondria were trapped by double-membrane vesicles in CSFV-infected cells, suggesting CSFV infection with CSFV-induced mitophagy. The Pink1/Parkin signaling pathway is one of the important mechanisms mediating the activation of mitophagy [76]. The authors also showed that the increased translocation of Pink1 and Parkin in purified mitochondria of CSFV-infected cells was observed. Meanwhile, CSFV upregulated the ubiquitination of MFN2 both in PK-15 and 3D4/2 cells. Among many mitochondrial membrane proteins, the ubiquitination degradation of the MFN2 protein has been proven to be closely related to the mitochondrial translocation of Parkin [77]. Further, PK-15 and 3D4/2 cells transfected with the GFP-LC3 plasmid were infected by CSFV and analyzed by confocal immunofluorescence assay. The data showed that the mitochondria conjunct with Parkin was trapped by GFP-LC3 puncta. The authors also utilized a tandem-tagged mRFP–GFP plasmid encoding a mitochondrial targeting signal sequence to assess mitophagy. This showed that CSFV-infected cells displayed greater red fluorescence protein (RFP) fluorescence, which indicated the degradation of mitochondria by lysosomes. Finally, the fusion of GFP–LC3, mitochondria, and lysosomes in PK-15 and 3D4/2 cells was analyzed by confocal microscopy, which proved that mitochondria wrapped by LC3 puncta were connected with lysosomes in CSFV-infected cells. In addition, these authors determined that CSFV N^pro^ expression, RNA replication, and virus titers in the cells silenced endogenous Drp1 or Parkin through shRNA knockdown experiments. The results explored that knocking down Drp1 or Parkin suppressed CSFV replication, suggesting that CSFV promotes viral replication through mitochondrial division and mitophagy.

These above works indicate that CSFV infection not only results in autophagy in host cells, but also utilizes autophagy mechanisms for viral replication and virion release. Autophagy may be a pathogenic mechanism of CSFV to facilitate persistent viral infection.

## 4. Cross-Talk between Apoptosis and Autophagy in CSFV Pathogenesis

Autophagy and apoptosis are two main biological processes in cells, and there are significant differences between them in terms of metabolic pathways, morphological detection, and interaction with viruses. However, there is accumulating evidence that autophagy and apoptosis are closely related in function [78,79,80]. The activation of apoptosis requires the induction of an autophagy pathway, and autophagy can also protect cells by inhibiting apoptosis, which is conducive to the parasitism of pathogens in cells [81]. Studies have also shown that calpain, a molecule downstream of the apoptotic signal, can inhibit autophagy by degrading autophagy-associated proteins [82]. Therefore, it is necessary to assess how autophagy and apoptosis are linked in the context of viral infection to reveal the pathogenic mechanism of the virus.

It has been found that there are three relationships between autophagy and apoptosis in the process of inducing cell death, including collaboration, antagonism, and promotion [83]. Gou et al. found that autophagy and apoptotic signals were increased in the spleen of CSFV-infected piglets [84]. Interestingly, these autophagy-positive and apoptosis-positive cells were mainly distributed around splenic corpuscles. More importantly, CSFV caused LC3-II positive cells (about 40%) in pig spleen tissues, which simultaneously presented as TUNEL-positive. In vivo, the majority of apoptotic cells were found to be uninfected with CSFV. Similarly, some CSFV-infected cells did not exhibit signs of autophagy, and some cells that showed signs of autophagy were negative for CSFV infection, which implied a relation between autophagy and apoptosis in the spleen of pigs infected by CSFV. During the in vitro infection of CSFV, Pei et al. found that rapamycin-induced autophagy promoted cellular proliferation after virus infection, whereas shRNA-mediated inhibition of autophagy induced apoptosis in virus-infected cells, indicating that apoptosis is inhibited by CSFV-induced autophagy and thus contributes to virus propagation and persistent infection [85]. Interestingly, these authors also showed that the inhibition of autophagy with shRNA-based depletion of the essential autophagy proteins BECN1 and LC3 increased the expression of proapoptotic molecules, including Bax, cleaved-caspase3, and cleaved-PARP, and decreased the expression of antiapoptotic molecule Bcl-2 by upregulating the level of interferon-alpha (IFN-α), interferon-beta (IFN-β), tumor necrosis factor-related apoptosis inducing ligand (TRAIL), and factor associated suicide (FAS), indicating that apoptosis mediated by type I interferon is inhibited by CSFV-induced autophagy. Further, the authors analyzed the relationship between autophagy and retinoic acid inducible gene-I (RIG-I)-like receptor (RLR) signaling in CSFV infection and demonstrated that the expression level of RIG-I and melanoma differentiation-associated gene 5 (MDA5) were upregulated by silencing the gene expression of endogenous LC3 and BECN1, and the increased levels of type I interferon in autophagy-impaired cells were downregulated by silencing the gene expression of endogenous RIG-I and MDA5 during CSFV infection, suggesting that CSFV promotes type I interferon-induced apoptosis by upregulating the RLR signal. Moreover, silencing the gene expression of endogenous RIG-I and MDA5 upregulated CSFV-induced autophagic activities and increased the yield and titer of CSFV progeny, indicating that the RLR signal negatively regulates CSFV-induced autophagy. This work indicated that CSFV-induced autophagy inhibits cell apoptosis by downregulating RLR signaling-mediated levels of type I interferon production. Similarly, Gou et al. silenced endogenous Drp1 or Parkin through shRNA knockdown experiments and showed that CSFV enhanced the apoptosis of cells depleted of mitochondrial fission or mitophagy [33]. These studies demonstrate that CSFV-induced autophagy inhibits apoptosis and may be an important mechanism for persistent viral infection and immune escape.

## 5. Pyroptosis in the Pathogenesis of CSFV

Pyroptosis is a new form of programmed cell death that relies on the activation of caspase-1 and is accompanied by the release of a large number of proinflammatory cytokines [26]. Pyroptosis is closely linked with infectious disease occurrence, development, and immune regulation and plays an extremely important role in antagonizing and eliminating pathogenic infections and endogenous dangerous signals [29,86]. The morphological characteristics, mechanisms of occurrence and action, and factors involved in pyroptosis are significantly different from those of other cell death modes, such as apoptosis and necrosis [87]. Various viruses and viral components can induce pyroptosis. These viruses and viral components activate inflammatory corpuscles such as NLRP3, NLRC4, and NLRP1 and then cleave pro-caspase-1 into an active form [88,89]. Caspase-1 cleaves the gasdermin D (GSDMD) protein to form N- (GSDMD-N) and C- (GSDMD-C) terminal GSDMD fragments, in which GSDMD-N causes cell membrane perforation and cell charring [90,91]. On the other hand, caspase-1 cuts proforms of IL-1β and IL-18 into their mature forms, which reactivate and aggregate immune cells and induce the synthesis and release of other inflammatory cytokines (such as IL-6, IL-22, and IL-33), chemokines, and adhesion molecules, forming a "cascade effect", thus expanding the inflammatory response [92,93].

CSF is characterized by high fever (≥40.5 °C) and multiple hemorrhages. The pathological damage caused by CSFV mainly includes vascular endothelial injury and a massive reduction of lymphocytes. Among these effects, damage to the vascular endothelium leads to an increase in vascular permeability, which in turn causes a series of inflammatory pathological syndromes. These series of syndromes suggest that the occurrence and development of CSF is closely related to a series of physiological, pathological, and immune response processes [12]. More importantly, the pathogenesis caused by CSFV infection is similar to that caused by a "cytokine storm", that is, the massive secretion disorder of related cytokines is closely related to disease progression [94,95]. The inflammatory pathological response is an important feature of CSFV infection, which plays a very important role in the pathogenesis of CSF [96]. 

Early researchers long believed that apoptosis is the main mechanism for lymphopenia syndrome during CSFV infection. However, pyroptosis has been shown to have several morphological characteristics similar to apoptosis, including damage to DNA, as well as positive annexin V and TUNEL staining [87]. Recently, Yuan et. performed animal experiments to investigate CSFV-induced pyroptosis in peripheral lymphoid organs [34]. The authors observed that CSFV infection can increase the proportion of TUNEL-positive cell frequencies in porcine peripheral lymphoid tissues. They also showed that CSFV infection promotes the cleavage of GSDMD to produce active GSDMD-N in the peripheral immune organs of pigs. Recent studies have shown that GSDMD cleavage is mediated by caspases, with the N-terminal fragments of this protein thereupon driving pyroptosis [90,91]. For further analysis, calcein AM/EthD-III staining was used to detect cell membrane damage in peripheral blood monocytes (PBMCs) with CSFV infection. The authors found that CSFV infection increased the proportion of cell membrane damage in PBMCs, and this depended on the activation of caspase-1. Consistently, CSFV infection also promotes the cleavage of GSDMD and stimulates IL-1β production in PBMCs. All of these data demonstrated that pyroptosis is involved in CSFV infection. In viral infection, the assembly of the NLRP3 inflammasome is an important mechanism for caspase-1 activation, pyroptosis, and inflammation [26,88]. Studies by Fan et al. showed that CSFV-infected porcine PBMCs caused the activation of an ATP-dependent K^+^ ion channel and active NLRP3 inflammasome assembly, which led to caspase-1 activation and subsequent maturation and secretion of IL-1β, resulting in an inflammatory response [97]. Additional studies have showed that inhibiting the activation of the NLRP3 inflammasome can promote CSFV replication. These findings suggest that CSFV infection promotes the activation of NLRP3 inflammasome-mediated pyroptosis. In addition, studies have reported that *human immunodeficiency virus type 1* (HIV-1) infection causes a large reduction in CD4^+^ T cells, precisely because HIV-1 infection induces pyroptosis. It is noteworthy that only a small number of CD4^+^ T-cells are infected with HIV-1 [98]. Most cell death is caused by pyroptosis in infected cells. After the cells are damaged, the contents are released in large quantities, causing inflammation. The occurrence of this induces the death of the surrounding cells, the so-called "bystanders", which in turn creates a vicious circle that eventually leads to a significant reduction in CD4^+^ T-cells. In previous studies, some CSFV-infected peripheral lymphoid organs also showed positive TUNEL staining for cells that were not infected with CSFV, which likely occurred due to identical mechanisms through which HIV-1 infection causes bystander CD4^+^ T-cell death. These works suggest that CSFV-induced pyroptosis is an immune defense mechanism against the virus invading the body.

## 6. Conclusions and Future Directions

**Figure 1:**
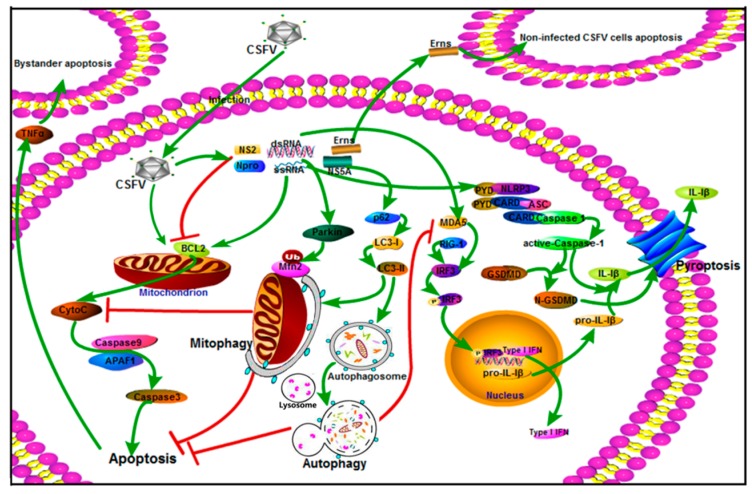
Apoptosis, autophagy, and pyroptosis in the pathogenesis of classical swine fever virus (CSFV). CSFV and its coding protein E^rns^ trigger apoptosis. The apoptosis of immune cells is beneficial for CSFV to escape the monitoring of the host immune system. Importantly, the CSFV nonstructural proteins N^pro^ and NS2 inhibit apoptosis, which may be an important process for the virus to achieve persistent infection. CSFV infection induces autophagy and mitophagy and utilizes their mechanisms for viral replication and virion release. Moreover, CSFV-induced autophagy inhibits cell apoptosis by downregulating retinoic acid inducible gene-I (RIG-I)-like receptor (RLR) signaling-mediated levels of type I interferon production, which may be an important mechanism for the immune escape of CSFV. CSFV infection promotes the activation of NLRP3 inflammasome-mediated pyroptosis, which may help explain why CSFV establishes a persistent infection in leukocytes.

Apoptosis, autophagy, and pyroptosis are the fundamental biological processes that maintain normal homeostatic and metabolic function in eukaryotic organisms, and they play an important role in antiviral immunity [23,24,25,26,27,28,29]. In this review, we discussed the molecular mechanisms of these three cellular biological processes and their prominent role in the pathogenesis of CSFV. As described above and as concluded in Figure 1, in the case of CSFV infection, the host initiates apoptosis, autophagy, and pyroptosis through different signaling pathways to mediate the antiviral immune response. However, CSFV has evolved a variety of strategies to regulate these three cellular biological processes and evade the host immune response, thus achieving persistent infection in the host. Apoptosis, as an effective mechanism for eliminating pathogens, can be triggered by CSFV and its coding protein E^rns^ [45,46]. However, the apoptosis of immune cells is beneficial for CSFV to escape the monitoring of the host immune system [31,43,44,45]. Importantly, CSFV nonstructural protein N^pro^ and NS2 have been shown to inhibit apoptosis, which may be an important process for the virus to achieve persistent infection [48,49,50]. Autophagy is generally considered to be a cell survival mechanism. However, during CSFV infection, CSFV utilizes autophagy mechanisms for viral replication and virion release [32,34]. Moreover, CSFV-induced autophagy inhibits cell apoptosis by downregulating RLR signaling-mediated levels of type I interferon production, which may be an important mechanism for the immune escape of CSFV [85]. Pyroptosis is another important biological process of the antiviral immune response. The activation of NLRP3 inflammasome-mediated pyroptosis during CSFV infection may help explain why CSFV establishes a persistent infection in leukocytes [34,97]. Generally, apoptosis, autophagy, and pyroptosis can be observed throughout the occurrence and development of CSFV, and they play key roles in ultimate decisions of CSFV-infected cells’ fates. Therefore, understanding the role of these three cellular biological processes in the pathogenesis of CSFV is important for the development of antiviral strategies. In recent years, although research on the interactions between CSFV and these three cellular biological processes has been deepening, the mechanisms are not yet fully clear. 

Recently, increasing numbers of studies related to apoptosis, autophagy, and pyroptosis pathways have contributed to a wealth of knowledge and facilitated a better understanding of virus pathogenesis [23,24,25,26,27,28,29]. On this basis, and in accordance with the pathogenic characteristics of CSFV and the known roles of apoptosis, autophagy, and pyroptosis in CSFV infection, future research should mainly focus on revealing the molecular mechanism of these three cellular biological processes in the immune regulation of CSFV infection from different levels and perspectives. Meanwhile, more work should be contributed to the in-depth excavation of key molecules in different types of these three cellular biological process signaling pathways, applying these target molecules to vaccine development or drug design to control CSFV infection and potentially treat disease. Apoptosis, autophagy, and pyroptosis are three main cellular biological processes with significant differences in their molecular mechanisms. However, these three cellular biological processes are not independent biological processes, but have potential interaction mechanisms [25,82,83,84]. Therefore, in future studies, it is necessary to further reveal the mechanistic interplay between apoptosis, autophagy, and pyroptosis in CSFV infection and immunity in order to reveal the pathogenesis and immune escape mechanism of CSFV, which will have important guiding significance for the development of specific drugs and vaccines for CSF prevention, control, and eradication.
